# Childhood Vascular Tumors

**DOI:** 10.3389/fped.2020.573023

**Published:** 2020-10-22

**Authors:** Harriet Bagnal Hinen, Luigi Boccuto, Cameron C. Trenor, Lara Wine Lee

**Affiliations:** ^1^Department of Dermatology and Dermatologic Surgery, Medical University of South Carolina, Charleston, SC, United States; ^2^College of Behavioral, Social and Health Sciences, Clemson University, Clemson, SC, United States; ^3^Division of Hematology/Oncology, Vascular Anomalies Center, Boston Children's Hospital, Boston, MA, United States

**Keywords:** hemangioma, pyogenic granuloma, pediatric vascular tumor, PHACE, angioma

## Abstract

Vascular tumors in pediatric patients are an important entity for the clinician to recognize and correctly diagnose. They may present at birth or develop at any point during infancy, childhood, or adolescence. Most are benign, but even benign lesions may have significant morbidity without proper intervention. Malignant vascular tumors are also rarely seen in the pediatric population, and may be associated with various syndromes.

## Introduction

Vascular tumors in pediatric patients are important for the clinician to be able diagnose, classify, and manage. They may present as a congenital lesion or develop at any point throughout infancy and childhood, and often follow a predictable clinical course depending on the type of tumor. The majority of vascular tumors occurring in children are benign, but even benign lesions may be associated with significant morbidity; it is important to be able to recognize the high-risk features associated with each type of tumor. The International Society for the Study of Vascular Anomalies (ISSVA) released updated classification guidelines for vascular anomalies in 2018; these guidelines divide vascular anomalies into vascular tumors (classified as benign, locally aggressive/borderline, and malignant) and vascular malformations (1). Vascular tumors will be further discussed in detail in this article.

## Discussion

### Infantile Hemangioma

Infantile hemangiomas are the most common benign tumor of childhood with a reported incidence of 4–5% in children <1 year of age (2). A female preponderance has been observed, with a female-to-male ratio of 3:1 to 5:1. This ratio is even higher in PHACES syndrome, with a female-to-male ratio reported up to 7:1 (3). There is also a higher risk associated with prematurity, multiple gestation pregnancy, and in infants born to mothers who underwent chorionic-villus sampling (3, 4). These vascular tumors are comprised of a benign proliferation of endothelial cells. Pathogenesis is unknown and likely multifactorial. The tumor cells stain positive for GLUT-1 protein throughout all stages of growth, which is not found in other vascular tumors. GLUT-1 is expressed in many tissues that serve as blood-tissue barriers including the placenta, brain, and retina (5). The tumors are considered benign, but they may exist in critical locations, and therefore be threatening to form or function. While mostly isolated in occurrence, association with other findings will be discussed.

Infantile hemangiomas follow a predictable clinical course comprised of a proliferative phase, a period of plateau or stability, followed by spontaneous regression. Up to 50% of patient's have a skin lesion present at birth, though this may be subtle clinically (5). They may present as telangiectases or erythematous macules and patches, often with a surrounding zone of pallor. The growth phase typically starts within the first month of life, with 80% of growth occurring within the first 5 months (6). Growth usually stops around 9–12 months of age. Most of these late-growth hemangiomas were classified as deep or mixed type hemangiomas (6). Growth after 36 months of age is rarely reported and more common in segmental hemangiomas of the head and neck and those involving deep and/or subcutaneous structures (7). Involution usually starts around 12–18 months of age, and can last for several years. Complete involution is predicted to occur at a rate of 10 percent per year, with the majority having completed involution by 5 years of age (5). It is important to note that complete involution does not imply normal skin left at the previous tumor site. Residual scarring, fibrofatty tissue, and telangiectases may persist (5). For this reason, it is important to determine the need for treatment early in the proliferative phase to prevent these sequelae in high risk lesions.

**Figure 1:**
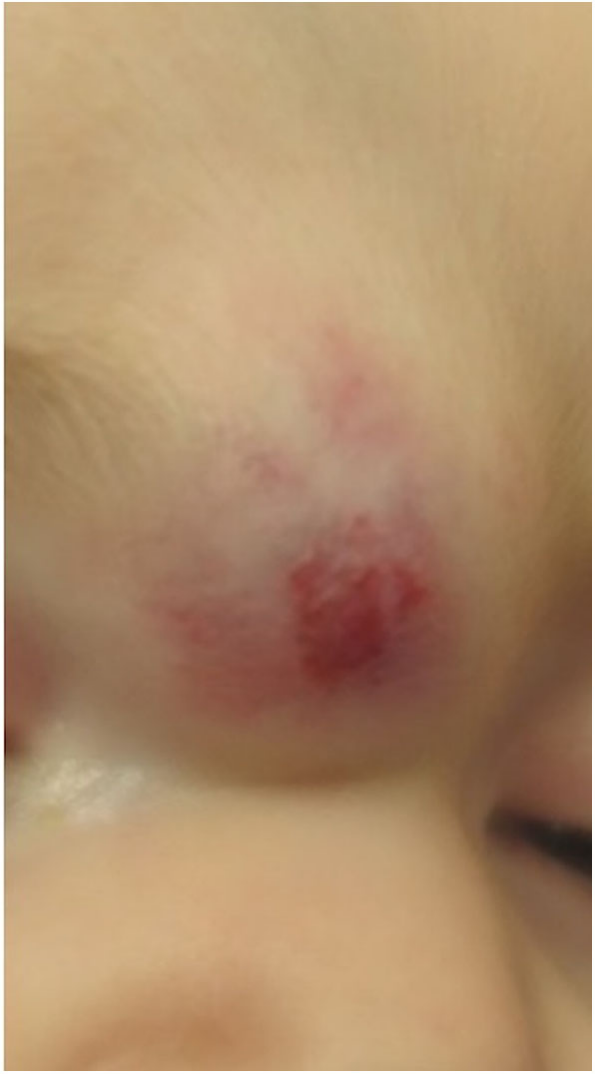
Compound infantile hemangioma of the glabella in a 5 month old infant.

**Figure 2:**
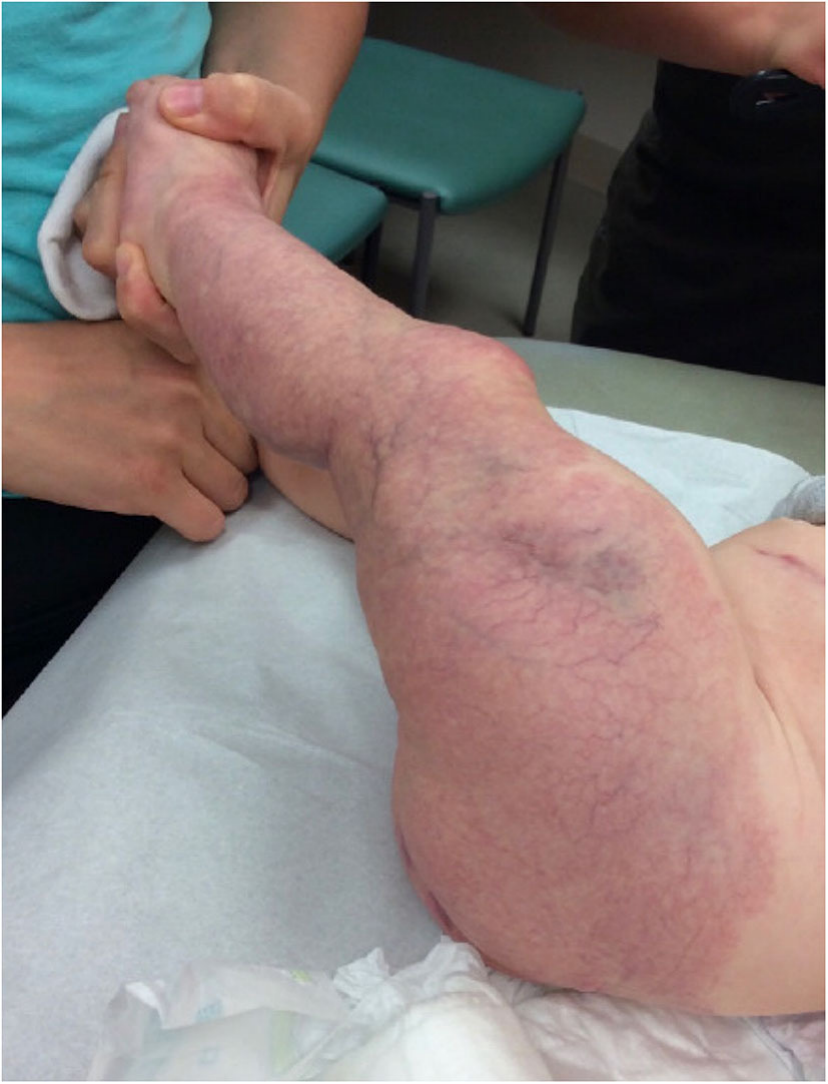
Extensive minimal growth infantile hemangioma (IH-Mag) of the lower extremity in an infant with LUMBAR syndrome.

Classification of an infantile hemangioma is based on the pattern (anatomic configuration) or type of lesion (depth) (1). Patterns include focal, multifocal, segmental, and indeterminant. Segmental lesions are determined in embryonic development and may be associated with various syndromes. Focal hemangiomas may be an isolated and innocuous finding, or could be threatening to function or life depending on the location. For example, the nasal tip/bridge, ear, periorbital, and lip are all concerning anatomic locations due to risk of impaired function or disfigurement. Additionally, intertriginous sites and lips are high risk for ulceration (2, 5). Infantile hemangiomas may also be classified by depth. Superficial lesions classically appear as bright red papules or plaques, while deep lesions are blue to violaceous nodules or tumors, sometimes with overlying telangiectases. Mixed lesions also exist, which have both superficial and deep components (Figure 1) (1). Infantile hemangiomas, most often segmental IH, may fail to fully proliferate and therefore retain the course telangiectatic appearance of a precursor lesion. These are termed minimal growth hemangioma or IH-MAG (Figure 2) (8).

**Figure 3:**
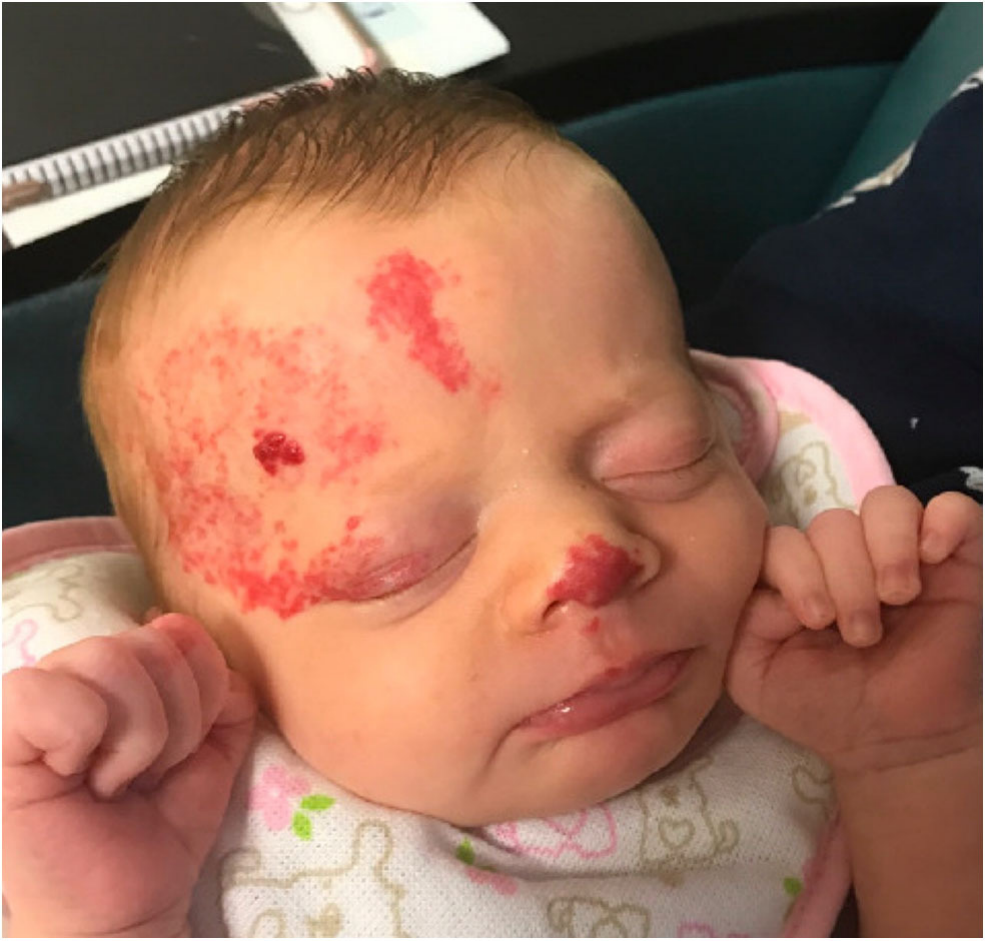
Facial infantile hemangioma in a 6 week old infant with PHACE syndrome.

Infantile hemangiomas with a segmental morphology must be given special consideration. Large segmental hemangiomas of the face and neck may be seen in PHACE syndrome, most commonly >22 cm^2^ (Figure 3) (9). Concomitant congenital anomalies in PHACE syndrome may include posterior fossa malformations, hemangiomas, arterial anomalies, coarctation of the aorta and cardiac defects, eye abnormalities, and sternal clefting or supraumbilical raphe (1, 10). Further evaluation is needed in patients suspected of having PHACE syndrome, including imaging and evaluations by neurology and ophthalmology. Vascular anomalies are the most common of the PHACE associations, and all patients with suspected PHACE should undergo MRI imaging of the cerebral vasculature. Based on these findings, patients should be risked stratified for risk of acute ischemic stroke and appropriate surveillance and intervention considered (11). Endocrinologic dysfunction has also been reported in PHACE syndrome including hypothyroidism, growth hormone deficiency, and pituitary dysfunction (12).

Another anatomic area of concern is the cervicofacial or “beard” distribution. Large or multifocal infantile hemangiomas in this area may be associated with airway involvement that can compromise respiratory function. One retrospective review found that 63 percent of patients with extensive infantile hemangiomas in this distribution had associated symptomatic airway involvement (13). Consideration of further imaging and referral to specialists should be given to these patients. Airway hemangiomas may occur in the absence of cutaneous hemangiomas.

Lumbosacral or extensive lower body segmental infantile hemangiomas are also lesions that may be associated with congenital anomalies (14). Multiple acronyms have been proposed to encompass findings associated with these hemangiomas including LUMBAR, SACRAL, and PELVIC syndromes (3). Extensive lower body hemangiomas with a minimal growth morphology were most commonly associated with LUMBAR syndrome. Associated findings include lower body hemangiomas, urogenital anomalies, ulceration, myelopathy, bony deformities, anorectal malformations, arterial anomalies, renal anomalies (1). Work-up including imaging should be guided by location of the hemangioma (14).

The majority of infantile hemangiomas occur in isolation, however approximately 20% of patients will have more than 1 lesion (4). If a patient has five or more cutaneous infantile hemangiomas involving any site, screening abdominal ultrasound should be performed to rule out the presence of hepatic hemangiomas. Hepatic hemangiomas can occur in three patterns; focal, diffuse, or multifocal. Focal lesions usually represent congenital hemangiomas, while diffuse and multifocal patterns are more classic for infantile hemangiomas (4, 15).

Multifocal hepatic infantile hemangiomas are usually asymptomatic, but may be associated with high-output cardiac failure due to vascular shunting. Diffuse hepatic infantile hemangiomas are higher risk, and may cause hepatomegaly resulting in abdominal compartment syndrome (4, 15). Both multifocal and diffuse patterns may also be associated with consumptive hypothyroidism due to the presence of intralesional type 3 iodothyronine deiodinase (16). Patients with symptomatic hepatic hemangiomatosis require prompt treatment with oral propranolol 2–3 mg/kg per day to avoid these life-threatening complications.

Several treatment options exist for infantile hemangiomas, and determination of therapy depends on multiple factors. A prospective study by Haggstrom et al. found that the most important predictors of poor outcomes associated with infantile hemangiomas are large size, segmental morphology, and facial location (17). Presence of ulceration is the most common complication, which may lead to scarring and pain.

The most common treatment for infantile hemangiomas is active observation, given the propensity for these lesions to completely regress. If a lesion is ulcerated or has high-risk features, other therapies should be considered. The first-line treatment for infantile hemangiomas requiring systemic therapy is oral propranolol 2–3 mg/kg/day, which has replaced systemic corticosteroids as the gold standard. If indicated, oral propranolol should be used for lesions throughout the entire proliferative stage. The medication may be initiated in the outpatient setting in infants older than 5–8 weeks corrected gestational age, without comorbid conditions. Heart rate and blood pressure should be monitored for upon initiation and with dose titrations (3). Extensive counseling with parents is required regarding potential side effects of propranolol, and they should be made aware of when to hold doses of the medication if needed. Additionally, doses should be given after a meal to prevent hypoglycemia (3). Hemangiomas falling in a high-risk category should have early referral to a hemangioma specialist for treatment initiation according to the AAP consensus guidelines (2).

Topical timolol and topical or intralesional corticosteroids may also be used as treatment for smaller, focal infantile hemangiomas. For ulcerated lesions, Pulse Dye Laser is a treatment option, though caution must be taken as this may induce ulceration of hemangiomas in the proliferative phase (3). Other therapies that have historically been used to treat infantile hemangiomas include interferon, vincristine, systemic corticosteroids, and cyclophosphamide, however these are now typically only used in rare circumstances for lesions resistant to treatment with propranolol. Systemic sirolimus has recently been successfully used to treat refractory hemangiomas, and is a promising emerging therapy for several vascular tumors (5, 18, 19). Embolization and surgical removal may also be an option, especially for larger, pedunculated lesions that are likely to heal with disfigurement. Finally, lasers are useful therapies both for lesions in the proliferative phase, as well as for treating sequalae in regressed lesions including telangiectases and scarring (5).

### Congenital Hemangiomas

**Figure 4:**
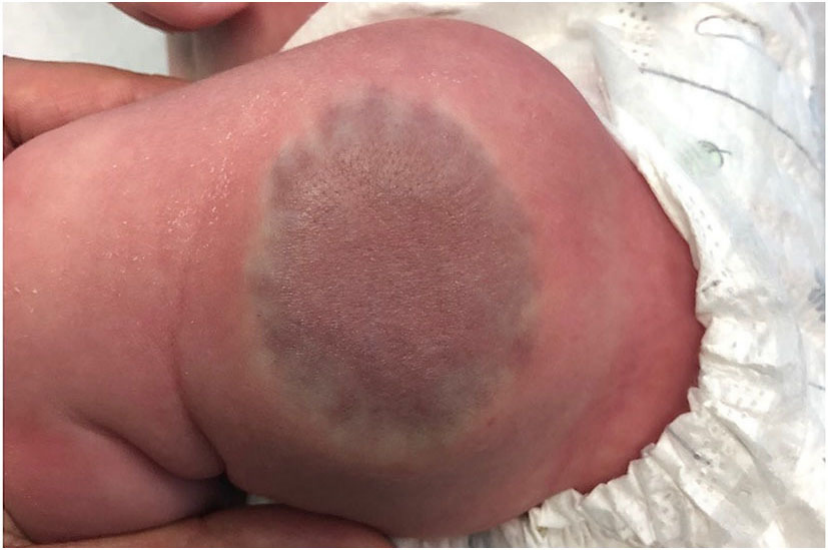
Rapidly involuting congenital hemangioma on the thigh of a 2 month old infant.

Congenital hemangiomas, unlike infantile hemangiomas, present fully formed at birth and may be diagnosed *in utero*. They are much more rare than infantile hemangiomas. There are three defined types; rapidly involuting congenital hemangiomas (RICH), partially-involuting congenital hemangiomas (PICH), and non-involuting congenital hemangiomas (NICH) (1). RICHs often present as exophytic masses that start to involute shortly after birth, and completely regress by 6–14 months of age (Figure 4). They may be associated with a localized consumptive coagulopathy and thrombocytopenia, though less severe than in Kasabach-Merritt Phenomenon, an entity discussed later in this article (3, 20). Residual atrophy and scarring is often found following regression. NICHs are often broad plaques, and less exophytic. They do not involute, and grow proportionately with the patient.

These lesions can be differentiated from infantile hemangiomas by the natural history, histology, and immunophenotype (21). Congenital hemangiomas are GLUT-1 negative, unlike infantile hemangiomas. Histologically they are comprised of lobules of proliferating capillaries that are separated by dense, abnormal fibrotic stroma. The overlying epidermis is atrophic and there is loss of dermal adnexal structures. This is unlike infantile hemangiomas, in which the proliferating lobules of capillaries are separated by normal connective tissue and overlying epidermis is not atrophic in non-regressed lesions (21). Somatic activating mutations in GNAQ and GNA11 have been identified in a subset of congenital hemangiomas (22).

Treatment of congenital hemangiomas depends upon multiple factors including the type, size, and location. Observation is often recommended for initial management, and the clinician may consider imaging or biopsy to confirm diagnosis if it is in question. For large exophytic RICHs, redundant atrophic tissue may persist after involution that may require surgical excision. Congenital hemangiomas may be associated with ulceration and life-threatening hemorrhage and thrombocytopenia. In addition, large congenital hemangiomas, particularly in the liver, may induce a high-output cardiac state. Resection is the only known treatment for complicated congenital hemangiomas. Surgery may also be required for large NICHs. Pulse dye laser can be used to treat superficial telangiectases (3).

### Pyogenic Granuloma

**Figure 5:**
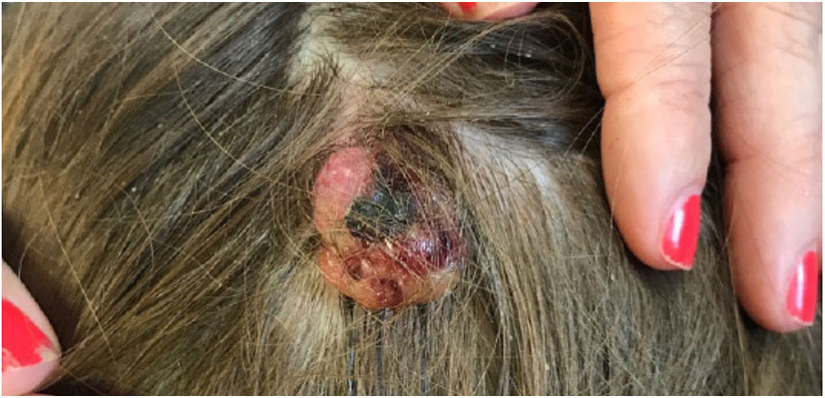
Large pyogenic granuloma on the scalp.

**Figure 6:**
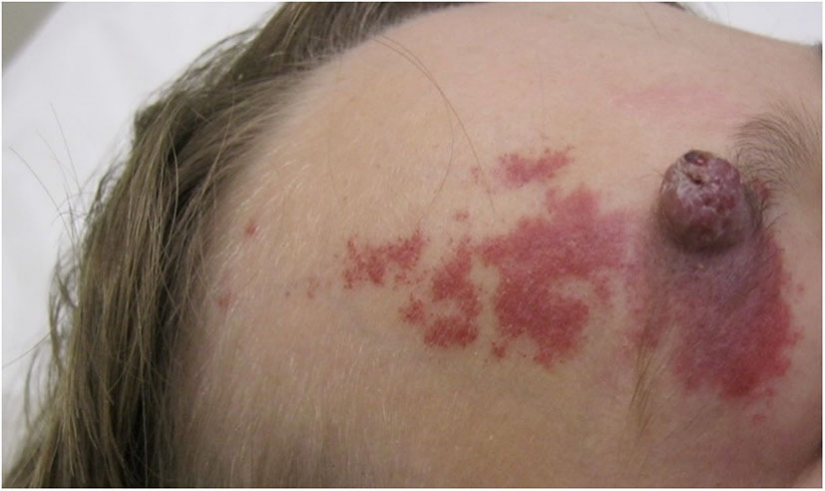
Pyogenic granuloma-like growth arising within a capillary malformation.

Pyogenic granulomas (PGs), also known as lobular capillary hemangiomas, are a common acquired benign vascular tumor. Clinically, these lesions present as red to brown papules that may have a collarette of scale and bleed easily when traumatized (Figure 5). They can occur anywhere, but most commonly occur on exposed areas of skin in sites of trauma including the hands, face, and mucous membranes. They are usually solitary, but may be multiple and agminated, as seen in association with pre-existing capillary malformations (Figure 6) (23). PGs may occur more frequently during pregnancy or in association with certain medications (24).

The treatment of choice for pyogenic granulomas is most often surgical excision followed by electrodessication or curettage of the base of the lesion to help prevent recurrence. Small lesions can also be treated with the Pulse Dye Laser or combined continuous-wave/pulsed CO_2_ laser to help minimize scarring and other adverse effects (25, 26). Topical imiquimod and topical or oral beta-blockers have also been successfully used as a non-invasive treatment option (27).

### Tufted Angioma

Tufted angiomas are classified as benign vascular tumors by the ISSVA, but it is pertinent to distinguish them from infantile and congenital hemangiomas, as they may be complicated by Kasabach-Merritt Phenomenon. Tufted angiomas typically appear within the first 5 years of life and may be present at birth, though sporadic cases of acquired tufted angiomas in adult patients have been reported (28, 29). They are slow-growing, erythematous to violaceous indurated plaques on the neck or upper trunk, often poorly-demarcated. Some lesions have been reported to have overlying hypertrichosis and hyperhidrosis. Histologically, they demonstrate tufts and lobules of capillaries in a cannonball pattern (28). Some consider tufted angiomas to be on a spectrum with kaposiform hemangioendotheliomas, as they often share similar histologic features and both can be associated with Kasabach-Merritt Phenomenon.

### Kaposiform Hemangioendothelioma

**Figure 7:**
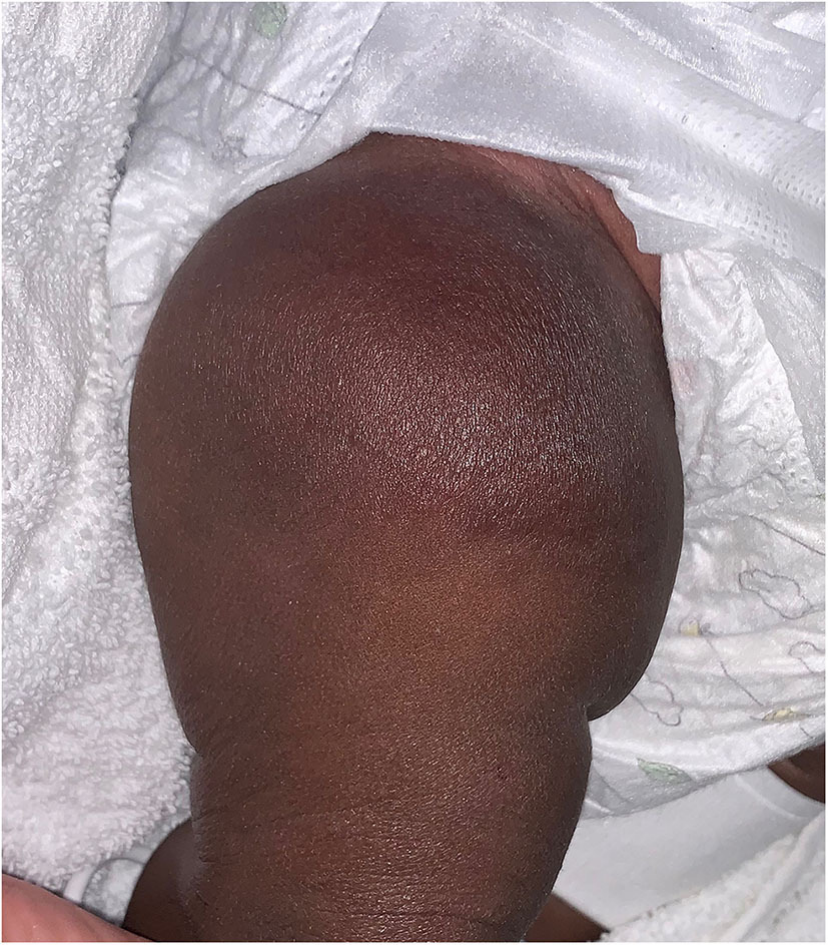
Kaposiform hemangioendothelioma on the thigh of an infant.

Kaposiform hemangioendotheliomas (KHE) are rare tumors that present in infancy or early childhood; they are classified as locally aggressive or borderline vascular tumors (1). KHEs may present as a rapidly expanding firm violaceous plaque in the skin, that often infiltrates deep soft tissue and bone (Figure 7). They may occur in the retroperitoneum as well as visceral locations, making diagnosis particularly challenging. Histologically, the lesions demonstrate some features similar to tufted angiomas, though they can be distinguished by the presence of lymphangiomatosis and a sheet-like pattern of growth that may resemble Kaposi's sarcoma (28). Kaposiform hemangioendotheliomas are also larger and less well-defined tumors than tufted angiomas. Prognosis depends on the extent and location of the tumor. Poor prognosis is associated with visceral disease and consumptive thrombocytopenia, known as Kasabach-Merritt Phenomenon (KMP). KMP is associated with ~70% of KHEs, and has a propensity to occurs in large lesions (>8 cm) that are located in the retroperitoneum or intrathoracic region (30, 31). Treatment of both KHE and tufted angioma is difficult, but management is primarily medical. Successful interventions have included systemic corticosteroids, cyclophosphamide, vincristine, and oral sirolimus. Sirolimus in particular is a promising emerging therapy for the medical management of these tumors. The first reported successful case of refractory KHE treated with sirolimus was in 2010 (32). Several studies published since that time have also showed promising results. A recent retrospective study by Wang et al. showed reduction in tumor size and normalization of platelet counts in 19 of 20 patients with KHE who completed therapy with oral Sirolimus. This study showed no evidence of recurrence after a median follow-up time of 32 months, and average time to response to therapy was 1 week (33). Though medical management predominates in the treatment of tufted angiomas and kaposiform hemangioendotheliomas, if a lesion is localized and well-circumscribed, surgery may be an option. Embolization can also be used to stabilize very large lesions until medical therapy can be initiated (34–36).

Kasabach-Merritt Phenomenon is characterized clinically by a consumptive coagulopathy resulting in thrombocytopenia and hypofibrinogenemia, that can be seen in patients with tufted angiomas and kaposiform hemangioendotheliomas (37). Clinically, it is characterized by rapid enlargement of the vascular tumor with ecchymosis. KMP shows a variable response to treatment; both IV vincristine (combined with antiplatelet therapies) and oral sirolimus have shown promising results, though they have not been compared in a study. The conventional standard treatment was previously systemic corticosteroids which may be used initially, but should not delay the initiation of sirolimus or vincristine if indicated (35, 37). Other therapies for these lesions have included embolization, surgical excision, pulse dye laser, low-dose aspirin, and radiation therapy. Each of these therapies has limitations, and have shown mixed results regarding safety and efficacy (28, 38, 39).

### Dabska Tumor

Papillary intralymphatic angioendotheliomas (PILA), also known as Dabska tumors, are rare vascular tumors that are most commonly found in children. They are categorized by the ISSVA as locally aggressive or borderline vascular tumors (1). The Dabska tumor was first described as a low-grade angiosarcoma in 1969 by Maria Dabska, who published a case series of six patients. Three of the six patients had lymph node involvement, and one patient had distant metastasis of the tumor resulting in death (40). Since it was first described, other cases have been reported that have seemingly behaved in a more benign manner (41). Histologically, the tumors are characterized by an intravascular proliferation of hobnail endothelial cells that form characteristic intraluminal papillary projections. They also have evidence of lymphatic vessels either histologically or immunophenotypically. Given the presence of these features histologically, the name Papillary Intralymphatic Angioendothelioma (PILA) was proposed by Fanburg-Smith et al. (41). Clinically, the tumor appears as a slow-growing violaceous to erythematous nodule or plaque, that over time may become more poorly-defined with palpable projections or satellite lesions. There is no predilection for gender or anatomic site (42). Treatment for these lesions is surgical excision with clear margins and close follow-up, given the potential for lymph node involvement and distant metastasis.

### Hemangioendothelioma

#### Epithelioid Hemangioendothelioma

Epithelioid hemangioma (EHE) is a rare malignant vascular tumor that has overlapping of features of both angiosarcoma and epithelioid hemangioma. The tumor most commonly results from a translocation between chromosomes 1 and 3 that creates a pathopneumonic WWTR1-CAMTA1 fusion protein. Less often, it may result from a YAP1-TFE3 fusion (43–45). EHE most often occurs in middle age, however pediatric cases have been reported. Clinically, EHE has a variable presentation and has been reported to affect many different organs. Liver involvement is the most common presenting body site to be involved (21%), followed by both liver and lung involvement (18%), then bone alone (14%), and then lung involvement alone (12%) (43, 44). EHE may also involve the subcutaneous fat, presenting as subcutaneous nodules. Affected patients may have systemic symptoms, such as weight loss, fatigue, and fever, however the malignancy is commonly asymptomatic and often diagnosed by incidental findings on chest imaging (43, 44, 46). Prognosis is variable; poor outcomes are associated with systemic symptoms, metastases at time of diagnosis, and increased mitoses on pathology (43, 46, 47). Management is variable and data is limited given the rarity of the malignancy. Treatment options may include chemotherapeutic agents, immunotherapy, and targeted therapies (43). Surgical resection is an option for localized disease, and watchful waiting may be considered for asymptomatic disease, as spontaneous regression has been reported (46). Liver transplant for patients with hepatic EHE has also been reported as a successful treatment option. Interestingly, the presence of lymph node or extra-heptic involvement did not impact disease free survival in a series of 59 patients with hepatic EHE treated with liver transplant (43, 48).

#### Pseudomyogenic Hemangioendothelioma

Pseudomyogenic hemangioendothelioma (PHE) is a recently recognized, locally aggressive or borderline vascular tumor (1). The tumor expresses a fusion gene between FOSB and either SERPINE1, ACTB, or WWTR1, which results in an overexpression of FOSB (49). PHE most frequently occurs in young adult males, and often presents as grouped nodules on the lower limb. Histologically, the tumor is composed of sheets and cords of spindled cells with eosinophilic cytoplasm. Despite the resemblance of myoid cells, the tumor cells stain negative for desmin and positive for endothelial markers (45, 49, 50). Treatment of PHE is often determined by the size and location of the tumor; surgical excision is usually the treatment of choice, but given the propensity for PHEs to be multifocal, surgery may not be an option. Additionally, one third of patients have recurrence after surgical excision (51). In such cases, medical management with gemcitabine, sirolimus, and everolimus have been used successfully (50–52). Given the rarity and recent discovery of PHE, clinical trials have not yet been conducted, so further research is needed in medical management treatment options.

#### Other Hemangioendothelioma

There are several other borderline or locally aggressive vascular tumors that are classified as hemangioendotheliomas. These include the retiform hemangioendothelioma, composite hemangioendothelioma, and polymorphous hemangioendothelioma (1). Each of these tumors has unique histopathologic findings that aids in diagnosis. Most are low-grade neoplasms that have the potential to metastasize, though they rarely do. They vary in aggressiveness, and often recur after excision. Treatment of hemangioendotheliomas is typically handled on a case-by-case basis, and depends on histologic features and clinical aggressiveness (53).

### Angiosarcoma

Angiosarcomas are uncommon, highly aggressive vascular tumors that usually present in the skin or soft tissue on the head and neck of elderly patients, but can affect any visceral organ. They are very rarely reported in children, and account for only 0.3% of pediatric sarcomas (54). The diagnosis portends a poor prognosis; angiosarcomas are often aggressive and have a tendency to metastasize (55). Known risk factors for developing angiosarcoma include long-standing lymphedema, prior radiation, and inherited familial syndromes including Neurofibromatosis Type I and Klippel-Trenaunay syndrome (54). Clinically, the tumor can present as an expanding bruise-like lesion, or as an erythematous to violaceous nodule or plaque. Visceral lesions often present as an expanding mass. Treatment of angiosarcoma is challenging, and recurrences are common. Successful therapies have included multi-agent cytotoxic chemotherapy, immunotherapy, tyrosine kinase inhibitors and propranolol, combined with surgical resection and radiation (56).

### Vascular Syndromes With Malignancy Risk

Beckwith-Wiedemann syndrome (BWS) is a vascular syndrome associated with other characteristic congenital anomalies, and affected patients have an increased risk of developing various malignancies. BWS results from mutations on chromosome 11p15.5 and may present with hemihyperplasia, centrofacial capillary malformation, macrocephaly, macroglossia, hypoglycemia, and organomegaly (57, 58). Patients with BWS are at increased risk of developing several embryonal malignancies including Wilms tumor, hepatoblastoma, rhabdomyosarcoma, and neuroblastoma. Risk of tumor development in affected patients is ~5–10 percent, with Wilms tumor being the most frequent tumor observed (58). Nephromegaly is considered to be a strong risk factor for developing Wilms tumor in these patients (59). The vast majority of the tumors in BWS occur intra-abdominally, therefore screening with abdominal ultrasound three to four times a year can be very useful in early detection and treatment of malignancies in these patients (58, 60, 61). Lapunzina et al. also suggests serial screening with physical examination, urinalysis, various serological tests, chest x-ray, and urine VMA, HVA, and catecholamines at varying intervals depending on age. As the majority of tumors are embryonal in origin, most malignancies occur in infancy or early childhood, so screening should be more frequent in younger patients.

CLOVES (congenital lipomatous overgrowth, vascular malformations, epidermal nevi, and skeletal anomalies) syndrome is another vascular syndrome that has an increased risk of malignancy. Affected patients have an increased risk of Wilms tumor that is reported to be similar to that seen in Beckwith-Wiedemann syndrome and other isolated hemihypertrophy disorders (57). CLOVES syndrome is caused by a postzygotic activating PIK3CA mutation, and is considered by many to be on a spectrum with other disorders characterized by PIK3CA somatic mutations. The PIK3CA-related overgrowth spectrum (PROS) disorders also include macrocephaly-capillary malformation, Klippel-Trenaunay syndrome (KTS), macrodactyly, isolated lymphatic malformation and others (57, 62). Outside of CLOVES syndrome, Wilms tumor has only been reported in 4 other patients with PIK3CA-related overgrowth spectrum (PROS) disorders including two cases seen in macrocephaly-capillary malformation (M-CM) (57, 62–64). Other PROS disorders, including Klippel-Trenaunay syndrome (KTS), have not been shown to be associated with an increased risk of Wilms tumor or other malignancy compared to the general population (57, 65, 66). Given the increased risk in patients with CLOVES syndrome and the benefit of early detection of Wilms tumor, these patients may benefit from screening ultrasounds. Peterman et al. proposes abdominal ultrasounds on a screening schedule similar to that for BWS; every 3 months until 7 years of age, with most tumors expected to be detected before 3 years of age (57).

Other syndromes with increased risk of malignancy include those with mutations in the PTEN tumor suppressor gene. Also known as PTEN hamartoma tumor syndromes (PHTS), these disorders include Cowden syndrome and Bannayan-Riley-Ruvalcaba syndrome. These disorders are allelic to one another, but have clinically distinct phenotypes (67). Cowden syndrome is usually diagnosed in adolescence or adulthood, and is characterized by pathopneumonic dermatologic findings including trichilimmomas and numerous papillomatous lesions of the skin and mucosa. Patients with Cowden syndrome have a significantly increased risk of several malignancies including breast, endometrial, and thyroid carcinomas (67). Patient's with Bannayan-Riley-Ruvalcaba syndrome are usually diagnosed in childhood. They also have an increased risk of tumors, but unlike Cowden syndrome, most of these tumors are benign and include lipomas, angiolipomas, and hamartomatous GI polyps. Other common clinical findings include penile lentigines and macrocephaly (58, 67). Vascular tumors can be seen in both Cowden Syndrome and Bannayan-Riley-Ruvalcaba syndrome; hemangiomas and arteriovenous malformations have both been reported (67).

## Conclusions

Infantile hemangioma is a common vascular tumor in infants, but not all benign vascular tumors are hemangiomas. Other vascular tumors in children are relatively rare and important to recognize, given difference in natural history, clinical prognosis, and treatment options. Though the vast majority of pediatric vascular tumors are benign and diagnosed clinically, it may be difficult to determine the diagnosis and predict risk of a particular lesion, so further imaging or biopsy for tissue diagnosis may be warranted. Once diagnosed, it is important for the clinician to recognize high risk features of each tumor, including anatomic risks, morphology, potential for co-existing congenital anomalies, coagulopathy, and malignant potential. Treatment of pediatric vascular tumors is often multi-disciplinary and is influenced heavily by individual risks and benefits. The options for medical therapies are actively evolving through genetic discoveries and compassionate use in selected patients.

## Author Contributions

HH, CT, and LW contributed to the writing of the manuscript. LW and CT supervised the project and provided clinical images. LB contributed to the conceptualization, design, and editing of the manuscript. All authors contributed to the article and approved the submitted version.

## Conflict of Interest

The authors declare that the research was conducted in the absence of any commercial or financial relationships that could be construed as a potential conflict of interest.
