# Corrigendum: Childhood Vascular Tumors

**DOI:** 10.3389/fped.2021.649610

**Published:** 2021-02-24

**Authors:** Harriet Bagnal Hinen, Luigi Boccuto, Cameron C. Trenor, Lara Wine Lee

**Affiliations:** ^1^Department of Dermatology and Dermatologic Surgery, Medical University of South Carolina, Charleston, SC, United States; ^2^College of Behavioral, Social and Health Sciences, Clemson University, Clemson, SC, United States; ^3^Division of Hematology/Oncology, Vascular Anomalies Center, Boston Children's Hospital, Boston, MA, United States

**Keywords:** hemangioma, pyogenic granuloma, pediatric vascular tumor, PHACE, angioma

Dr. Luigi Boccuto was not included as an author in the published article. The corrected Author Contributions Statement appears below.

HH, CT, and LW contributed to the writing of the manuscript. LW and CT supervised the project and provided clinical images. LB contributed to the conceptualization, design, and editing of the manuscript. All authors contributed to the article and approved the submitted version.

The authors apologize for this error and state that this does not change the scientific conclusions of the article in any way. The original article has been updated.

